# Culprit Lesion Coronary Intervention Before Complete Angiography in ST-Elevation Myocardial Infarction: A Randomized Clinical Trial

**DOI:** 10.1001/jamanetworkopen.2024.3729

**Published:** 2024-03-29

**Authors:** Nir Levi, Rafael Wolff, Rami Jubeh, Mony Shuvy, Yoed Steinmetz, Nimrod Perel, Tomer Maller, Itshak Amsalem, Rafael Hitter, Elad Asher, Anna Turyan, Mohammad Karmi, Amir Orlev, Dmitry Dratva, Zahi Khoury, Tal Hasin, Arik Wolak, Michael Glikson, Danny Dvir

**Affiliations:** 1 Jesselson Integrated Heart Center, The Eisenberg R&D Authority, Shaare Zedek Medical Center, Faculty of Medicine, The Hebrew University of Jerusalem, Jerusalem, Israel; 2 Heart Institute, Ha’Emek Medical Center, Faculty of Medicine, Technion-Israel Institute of Technology, Haifa, Israel

## Abstract

**Importance:**

Rapid reperfusion during primary percutaneous coronary intervention (PCI) is associated with improved outcomes among patients with ST-elevation myocardial infarction (STEMI). Although attempts at reducing the time from STEMI diagnosis to arrival at the catheterization laboratory have been widely investigated, intraprocedural strategies aimed at reducing the time to reperfusion are lacking.

**Objective:**

To evaluate the effect of culprit lesion PCI before complete diagnostic coronary angiography (CAG) vs complete CAG followed by culprit lesion PCI on reperfusion times among patients with STEMI.

**Design, Setting, and Participants:**

This open-label, prospective, randomized clinical trial was conducted between April 1, 2021, and August 31, 2022, among patients admitted to a tertiary center in Jerusalem, Israel, with a diagnosis of STEMI undergoing primary PCI. All patients were followed up for 1 year. Analysis was on an intention-to-treat basis.

**Intervention:**

Patients were randomized in a 1:1 ratio to undergo either culprit lesion PCI before complete CAG or complete CAG followed by culprit lesion PCI.

**Main Outcomes and Measures:**

A needle-to-balloon time of 10 minutes or less.

**Results:**

A total of 216 patients were randomized, with 184 patients (mean [SD] age, 62.9 [12.2] years; 155 men [84.2%]) included in the final intention-to-treat analysis; 90 patients (48.9%) were randomized to undergo culprit lesion PCI before CAG, and 94 (51.1%) were randomized to undergo to CAG followed by PCI. Patients who underwent culprit lesion PCI before complete CAG had a shorter mean (SD) needle-to-balloon time (11.4 [5.9] vs 17.3 [13.3] minutes; *P* < .001). The primary outcome of a needle-to-balloon time of 10 minutes or less was achieved for 51.1% of patients (46 of 90) who underwent culprit lesion PCI before CAG and for 19.1% of patients (18 of 94) who underwent complete CAG followed by culprit lesion PCI (odds ratio, 4.4 [95% CI, 2.2-9.1]; *P* < .001). Rates of adverse events were similar between groups. In a subgroup analysis, the effect of culprit lesion PCI before complete CAG on the primary outcome was consistent. There were no differences in rates of in-hospital, 30-day, and 1-year all-cause mortality.

**Conclusions and Relevance:**

In this randomized clinical trial of patients with STEMI, culprit lesion PCI before complete CAG resulted in shorter reperfusion times. Larger trials are needed to validate these results and to evaluate the effect on clinical outcomes.

**Trial Registration:**

ClinicalTrials.gov Identifier: NCT05415085

## Key Points

**Question:**

What is the effect of culprit lesion percutaneous coronary intervention (PCI) before complete diagnostic coronary angiography (CAG) compared with CAG followed by culprit lesion PCI on reperfusion times among patients with ST-elevation myocardial infarction (STEMI)?

**Findings:**

In this randomized clinical trial that included 184 adults with STEMI, culprit lesion PCI before complete diagnostic CAG during primary PCI resulted in shorter reperfusion times and a statistically significant higher proportion of patients who achieved the primary outcome of a needle-to-balloon time of 10 minutes or less (51.1% vs 19.1%).

**Meaning:**

These findings suggest that culprit lesion PCI before complete diagnostic CAG is an effective method to shorten reperfusion times among patients with STEMI.

## Teaser

This randomized clinical trial assesses the effect of culprit lesion percutaneous coronary intervention before coronary angiography vs coronary angiography followed by culprit lesion percutaneous coronary intervention on reperfusion times for patients with ST-elevation myocardial infarction.

## Introduction

Primary percutaneous coronary intervention (PCI) is the preferred reperfusion strategy for patients presenting with ST-elevation myocardial infarction (STEMI).^1,2^ Previous studies have demonstrated the association between prompt primary PCI and improved outcomes for patients with STEMI and showed that even a relatively small reduction in the door-to-balloon time resulted in improved survival.^3,4,5,6^ Current guidelines of the European Society of Cardiology and American College of Cardiology/American Heart Association for the management of patients with STEMI recommend that primary PCI be performed within 60 or 90 minutes, respectively.^1,2^ To enhance the quality of care for patients with STEMI, the door-to-balloon time has evolved into a quality measure in many health care systems.^7^ In addition, structured key care processes aimed at reducing the time to reperfusion for patients with STEMI have been evaluated.^8^ However, most of these studies focused mainly on reducing the time from STEMI diagnosis to arrival at the catheterization laboratory and not on intraprocedural strategies aimed at shortening reperfusion time.^9,10^

In previous retrospective studies, culprit lesion PCI before complete diagnostic coronary angiography (CAG) was shown to shorten the reperfusion time for patients with STEMI.^11,12,13,14^ Another prospective study with short-term follow-up showed that using a single transradial guiding catheter in conjunction with culprit lesion PCI before CAG reduced the door-to-balloon time.^15^ The present study aimed to prospectively evaluate the effect of culprit lesion PCI before complete diagnostic CAG on reperfusion time, in-hospital outcomes, and long-term outcomes among patients with STEMI who undergo primary PCI.

## Methods

### Study Design and Population

This is a single-center, open-label, prospective, randomized clinical trial. The study included patients with a diagnosis of STEMI who underwent primary PCI between April 1, 2021, and August 31, 2022. Exclusion criteria included a history of coronary artery bypass graft (CABG) surgery and arrival at the catheterization laboratory with cardiogenic shock, requiring cardiopulmonary resuscitation, or receiving extracorporeal membrane oxygenation. In addition, patients for whom PCI was not performed for any reason (eg, normal coronary arteries, nonsignificant coronary artery disease [CAD], need for urgent CABG, and spontaneous coronary artery dissection) were excluded from the analysis. This study complies with the Declaration of Helsinki^16^ and was approved by the institutional review board at the Shaare Zedek Medical Center, which waived informed consent because there was a chance that reperfusion would be further delayed until a laborious effort was made to obtain the patients’ informed permission and unintentionally affect study results; furthermore, the institutional review board recognized that a CAG before PCI approach is not supported by evidence and is often not used in accordance to the operator decision. This trial was not funded by any external source. This study was prospectively registered in Clinical Trials.gov (NCT05415085). This study followed the Consolidated Standards of Reporting Trials (CONSORT) reporting guideline.

### Study Protocol and End Point

The full study protocol and statistical analysis plan are shown in Supplement 1. Patients with STEMI were randomized in a 1:1 ratio to undergo either culprit lesion PCI before CAG (starting with a guiding catheter followed by angiography of the contralateral coronary system with a diagnostic catheter) or to complete CAG followed by culprit lesion PCI (starting with a diagnostic catheter to the contralateral coronary system followed by a PCI to the culprit lesion with a guiding catheter). On patients’ arrival at the catheterization laboratory, sealed envelopes were opened by the catheterization laboratory technician who then informed the nurse and the operator regarding the patient’s allocation and appropriate study protocol. The diagnosis of STEMI was based on results of the electrocardiography performed in the emergency department or by the local emergency medical services and was in accordance with the electrocardiographic criteria of the Fourth Universal Definition of Myocardial Infarction.^17^ The suspected culprit artery was determined by the interventional cardiologist based on results of the electrocardiography. For patients with anterior or lateral STEMI, the suspected culprit artery was determined to be in the left coronary system, while for patients with an inferior STEMI, an algorithm-based approach was used to determine the suspected culprit artery (ie, right coronary artery vs left circumflex) (eFigure in Supplement 2).^18^ Six-French radial and femoral introducers sheaths were used for arterial access. Five-French catheters were used for diagnostic coronary angiography, and 6-Fr guiding catheters were used for PCI.

The primary outcome of this study was a needle-to-balloon time of 10 minutes or less. Secondary outcomes included the need for hemodynamic support (ie, mechanical and/or medical) during PCI, the need for invasive or noninvasive ventilation during PCI, and the rate of failed PCI.

### Data Collection

Baseline characteristic data including patient demographic data, medical history, and baseline medical treatment were collected from patients’ medical records. Procedural times including door time (ie, time of patient arrival to the hospital), start-case time (ie, time of patient arrival to the catheterization laboratory), needle time (ie, time of first attempt of arterial puncture), balloon time (ie, time of first balloon inflation in the culprit artery), and end-case time (ie, time of patient leaving the catheterization laboratory) were recorded by a catheterization laboratory technician. All cardiac catheterization films were reviewed by a board-certified cardiologist to validate study data and protocol adherence. Procedural data including access site, crossover from a radial to femoral approach, angiographic findings and CAD severity, coronary interventions performed, vital signs during the procedure, Thrombolysis in Myocardial Infarction (TIMI) flow grade before and after PCI, total fluoroscopy time, and contrast administered were retrieved from the catheterization laboratory report. Data regarding intraprocedural and postprocedural course, including the need for invasive and noninvasive ventilation, the need for inotropic and mechanical circulatory support, ventricular arrhythmias, stent thrombosis, mechanical complications, in-hospital mortality, and long-term mortality, were retrieved from patients’ medical records and the Israeli Population Registry. Left ventricular ejection fraction assessment, grade of mitral regurgitation, and the existence of left ventricular thrombus were determined based on results of predischarge echocardiographic examination.

### Power Calculation

Based on previously published data, the primary outcome of a needle-to-balloon time of 10 minutes or less was used for sample size calculation.^11,12,13,14^ The calculated sample size was a minimum of 50 patients per group to detect a 50% relative increase in the proportion of patients who achieved the primary outcome (power = 0.9, α = 0.05, and β = 0.1).

### Statistical Analysis

Characteristics were described as numbers and percentages for categorical variables and by mean (SD) or median (IQR) values for continuous variables. Relations between categorical variables were evaluated by the χ^2^ test and the Fisher exact test. The effect of categorical variables on continuous measurements was tested by use of the *t* test and the Mann-Whitney test. The choice of a parametric or nonparametric test depended on the distribution of a continuous variable. To assess the consistency of the effect of culprit lesion PCI before CAG on the primary outcome, we performed a post hoc subgroup analysis of 17 subgroups. The subgroup analysis was conducted using a univariate logistic regression model, fitting separate models for each subgroup to assess the association between the primary outcome and a single variable. All tests were 2-sided, and *P* < .05 was considered statistically significant. Analyses were performed using SPSS Statistics for Windows, version 25.0 (IBM Corp).

## Results

### Study Population

**Figure 1.**
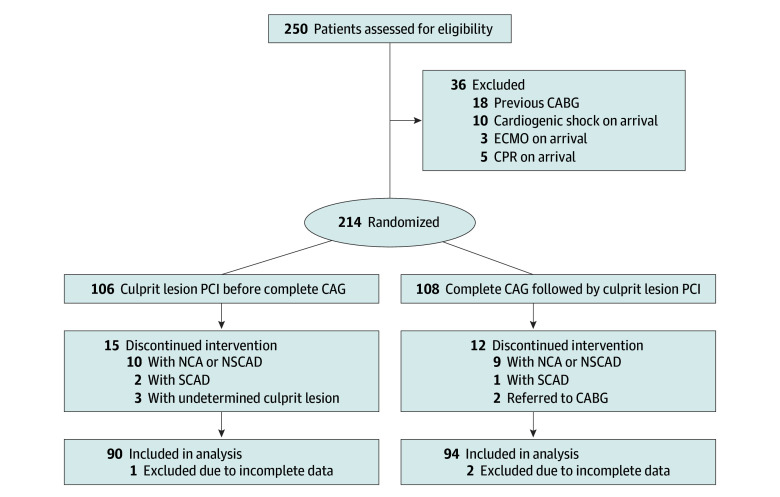
Participant Recruitment Flowchart CABG indicates coronary artery bypass graft; CAG, coronary angiography; CPR, cardiopulmonary resuscitation; ECMO, extracorporeal membrane oxygenation; NCA, normal coronary arteries; NSCAD, nonsignificant coronary artery disease; PCI, percutaneous coronary intervention; and SCAD, spontaneous coronary artery dissection.

Between April 1, 2021, and August 31, 2022, 214 patients with STEMI met the inclusion criteria and were randomized to undergo either culprit lesion PCI before CAG (106 patients) or complete diagnostic CAG followed by PCI to the culprit lesion (108 patients). Sixteen patients who underwent culprit lesion PCI before CAG and 14 patients who underwent complete CAG followed by PCI were excluded for various reasons. Hence, 184 patients (mean [SD] age, 62.9 [12.2] years; 155 men [84.2%]) were included in the final intention-to-treat analysis; 90 of 184 patients (48.9%) underwent culprit lesion PCI before CAG, and 94 of 184 patients (51.1%) underwent complete diagnostic CAG followed by PCI (Figure 1). The suspected culprit artery was assessed correctly for 168 patients (91.3%); however, for 9 of 90 patients (10.0%) in the culprit lesion PCI before CAG group and for 7 of 94 patients (7.4%) in the complete CAG followed by PCI group, the study protocol was not followed due to an incorrect assessment of the culprit artery; moreover, for only 2 of 90 patients (2.2%) in the former group, a culprit lesion could not be identified even after complete CAG. Additional baseline characteristics are detailed in Table 1.

**Table 1.** Baseline Patient Characteristics

Characteristic	Patients, No (%)	
	Culprit lesion PCI before CAG (n = 90)	CAG followed by culprit lesion PCI (n = 94)
Age, mean (SD), y	62.6 (11.4)	63.1 (13)
Sex		
Male	77 (85.6)	78 (83.0)
Female	13 (14.4)	16 (17.0)
Diabetes	32 (35.6)	26 (27.7)
Hypertension	39 (43.3)	43 (45.7)
Dyslipidemia	40 (44.4)	47 (50.0)
Smoker	42 (46.7)	41 (43.6)
Family history of CAD	11 (12.2)	14 (14.9)
BMI, mean (SD)	28.3 (4.9)	28.2 (5.0)
Congestive heart failure	2 (2.2)	2 (2.1)
Chronic renal failure	2 (2.2)	4 (4.3)
Peripheral vascular disease	1 (1.1)	4 (4.3)
Prior coronary artery disease	21 (23.3)	22 (23.4)
Prior PCI	21 (23.3)	18 (19.1)
Prior stroke	4 (4.4)	3 (3.2)
Baseline medications		
Aspirin	25 (27.8)	22 (23.4)
P2Y12 inhibitor	4 (4.4)	2 (2.1)
DOAC	2 (2.2)	6 (6.4)
β-Blocker	18 (20.0)	19 (20.2)
ACE inhibitor	18 (20.0)	20 (21.3)
ARB	5 (5.6)	6 (6.4)
Statin	36 (40.0)	28 (29.8)
Mode of presentation		
EMS	59 (65.6)	62 (66.0)
ED	31 (34.4)	32 (34.0)
Type of myocardial infarction		
Anterior and/or lateral	42 (46.7)	48 (51.1)
Inferior and/or posterior	47 (52.2)	46 (48.9)
Undetermined	1 (1.1)	0
Baseline TIMI flow grade, mean (SD)	1.3 (0.5)	1.4 (0.5)
Post-PCI TIMI flow grade, mean (SD)	2.8 (0.5)	2.7 (0.6)
First hs-cTnI level, median (IQR), pg/mL	258.5 (40.7-835.2)	91 (24-966.5)
Maximal hs-cTnI level, median (IQR), pg/mL	54 347 (25 673-111 303)	47 284 (16 217.2-122 544.7)

Abbreviations: ACE, angiotensin converting enzyme; ARB, angiotensin receptor blocker; BMI, body mass index (calculated as weight in kilograms divided by height in meters squared); CAD, coronary artery disease; CAG, coronary angiography; DOAC, direct oral anticoagulant; ED, emergency department; EMS, emergency medical services; hs-cTnI, high-sensitivity cardiac troponin I; PCI, percutaneous coronary intervention; TIMI, Thrombolysis in Myocardial Infarction.

### Procedural Characteristics

In both groups, most patients underwent the procedure through the radial approach. A crossover from a radial to a femoral approach was more common among patients who underwent CAG followed by culprit lesion PCI (5.6% [5 of 90] vs 14.9% [14 of 94]; *P* = .04). There were no significant differences between the groups in CAD severity and culprit artery location, but for 2 patients who underwent culprit lesion PCI before CAG, the culprit artery could not be determined. The pre- and post-PCI TIMI flow grades, number of stents deployed, amount of contrast injected, and fluoroscopy time were similar in both groups. Patients who underwent culprit lesion PCI before CAG had a higher mean (SD) arterial pressure at the end of the procedure (93.6 [16.6] vs 87.6 [18.9] mm Hg; *P* = .04).

### Primary and Secondary Outcomes

**Figure 2.**
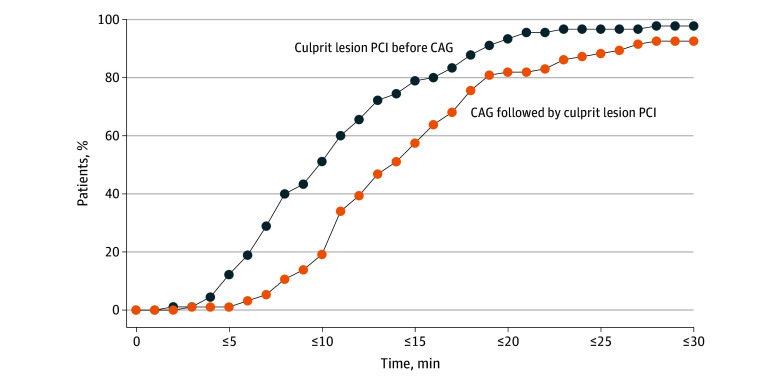
Cumulative Needle-to-Balloon Time CAG indicates coronary angiography; PCI, percutaneous coronary intervention.

**Figure 3.**
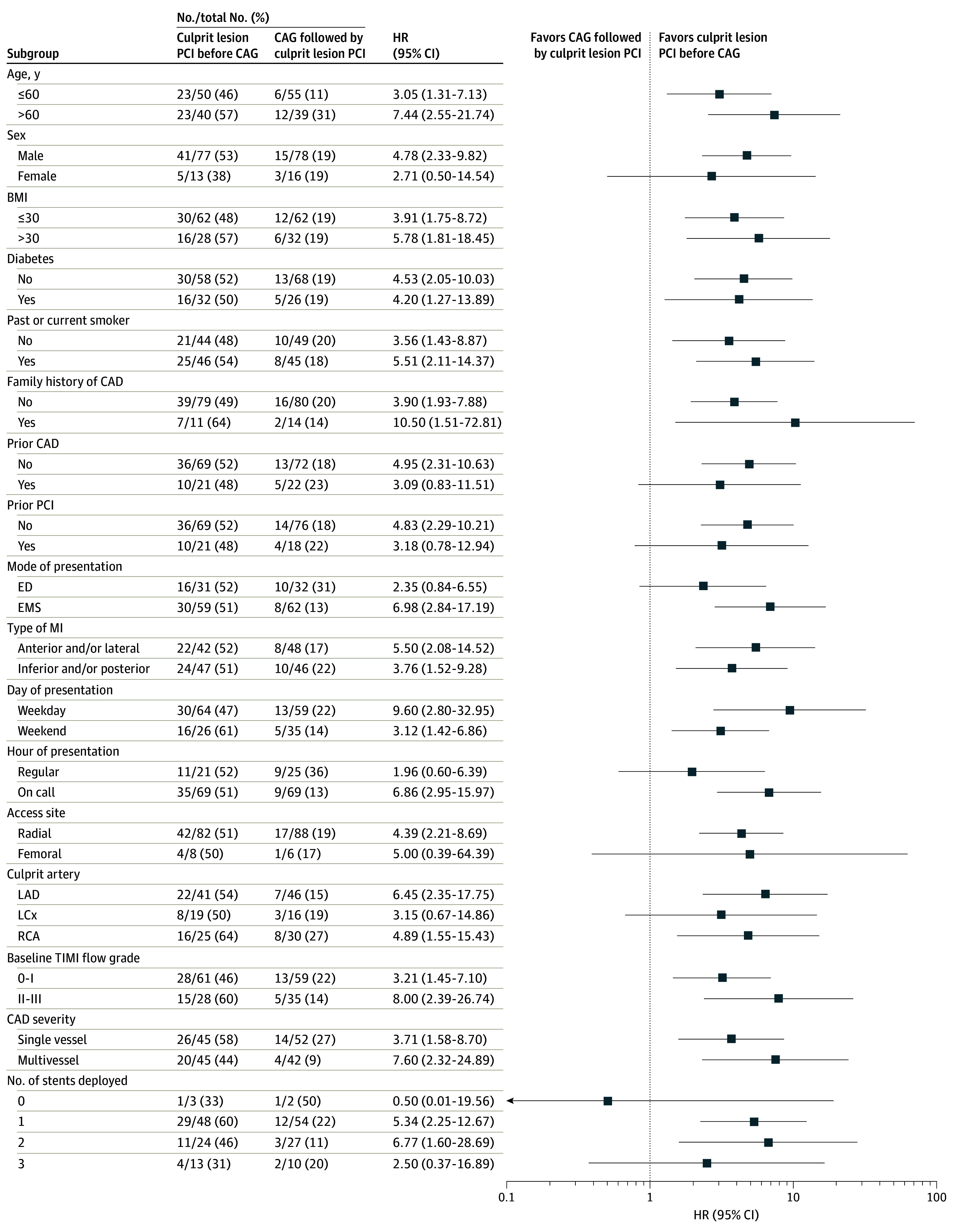
Subgroup Analysis for the Primary Outcome The primary outcome of the study, a needle-to-balloon time of 10 minutes or less, is shown according to subgroups. BMI indicates body mass index (calculated as weight in kilograms divided by height in meters squared); CAD, coronary artery disease; CAG, coronary angiography; ED, emergency department; EMS, emergency medical services; LAD, left anterior descending; LCx, left circumflex; MI, myocardial infarction; PCI, percutaneous coronary intervention; RCA, right coronary artery; and TIMI, Thrombolysis in Myocardial Infarction.

The primary outcome of a needle-to-balloon time of 10 minutes or less was achieved in 46 of the 90 patients (51.1%) who underwent culprit lesion PCI before CAG and in 18 of the 94 patients (19.1%) who underwent CAG followed by culprit lesion PCI (odds ratio, 4.4 [95% CI, 2.2-9.1]; *P* < .001). Patients who underwent culprit lesion PCI before CAG achieved reperfusion at all time points assessed (Figure 2). In addition, they had a shorter mean (SD) needle-to-balloon time (11.4 [5.9] vs 17.3 [13.3] minutes; *P* < .001), with no differences in the door-to-needle and total procedure times. There was no significant difference in the mean (SD) door-to-balloon time (61.6 [45.8] vs 73 [59.8] minutes; *P* = .12). In a subgroup analysis, the effect of culprit lesion PCI before CAG on the primary outcome was generally consistent across most subgroups but not among patients in whom the culprit lesion was in the left circumflex and those who underwent the procedure through femoral access (Figure 3). Rates of secondary outcomes, including vasopressor administration, need for mechanical circulatory support, and invasive or noninvasive ventilation during the procedure, were similar between the groups (Table 2).

**Table 2.** Postprocedural Course

Characteristic	Patients, No (%)		*P* value
	Culprit lesion PCI before CAG (n = 90)	CAG followed by culprit lesion PCI (n = 94)	
Acute stent thrombosis	1 (1.1)	4 (4.3)	.20
Any ventricular arrhythmia	20 (22.2)	16 (17.0)	.37
Need for direct current cardioversion	1 (1.1)	2 (2.1)	.52
Any hemodynamic and/or respiratory support	14 (15.6)	16 (17.0)	.79
Noninvasive ventilation	6 (6.7)	6 (6.4)	.94
Mechanical ventilation	2 (2.2)	6 (6.4)	.15
Vasopressor administration	11 (12.2)	12 (12.8)	.91
Mechanical circulatory support	3 (3.3)	5 (5.3)	.38
Predischarge echocardiography			
LVEF, mean (SD), %	44.2 (8.5)	42.6 (8)	.20
LVEF <40%	27 (30.0)	37 (39.4)	.18
Left ventricular apical thrombus	6 (6.7)	12 (12.8)	.16
≥Moderate MR	11 (12.2)	5 (5.3)	.17
Length of ICCU stay, mean (SD), d	2.6 (2.3)	3.2 (4.6)	.25
Total length of hospital stay, mean (SD), d	7.3 (25.1)	5.7 (7.3)	.52
In-hospital all-cause mortality	4 (4.4)	4 (4.3)	.61
30-d All-cause mortality	4 (4.4)	4 (4.3)	.61
365-d All-cause mortality	4 (4.4)	5 (5.3)	.53

Abbreviations: CAG, coronary angiography; ICCU, intensive coronary care unit; LVEF, left ventricular ejection fraction; MR, mitral regurgitation; PCI, percutaneous coronary intervention.

Six of the 90 patients (6.7%) who underwent culprit lesion PCI before CAG and 2 of the 94 patients (2.1%) who underwent CAG followed by culprit lesion PCI had a coronary dissection during the procedure, with no effect on outcome (*P* = .13). Two of the 90 patients (2.2%) who underwent culprit lesion PCI before complete CAG and 1 of the 94 patients (1.1%) who underwent complete CAG followed by culprit lesion PCI had coronary perforation (*P* = .53). A failed PCI, defined as a state of no reflow at the end of the procedure, occurred in none of the patients who underwent culprit lesion PCI before CAG and in 3 of the 94 patients (3.2%) who underwent complete CAG followed by culprit lesion PCI (*P* = .09). One of the 90 patients (1.1%) who underwent culprit lesion PCI before CAG and 4 of the 94 patients (4.3%) who underwent CAG followed by culprit lesion PCI had acute stent thrombosis. Predischarge echocardiographic parameters were similar in both groups. There were no significant differences in the length of stay in the intensive coronary care unit or in the total length of hospital stay. Eight patients died during hospitalization, 4 in each group. There were no differences in rates of 30-day and 1-year all-cause mortality. Additional details on the postprocedural course are detailed in Table 2.

## Discussion

In this randomized clinical trial, patients who underwent culprit lesion PCI before complete CAG were 4 times more likely to achieve the primary end point of a needle-to-balloon time of 10 minutes or less. This strategy was not associated with greater rates of intraprocedural or postprocedural adverse events or higher short- or long-term mortality rates. Moreover, patients who underwent culprit lesion PCI before CAG had a lower rate of crossover from a radial to femoral approach and higher mean arterial pressure at the end of the procedure. According to the results of the present study, culprit lesion PCI before complete CAG among patients with STEMI during primary PCI is safe and leads to a shorter reperfusion time.

Previous studies have demonstrated that even among patients with a door-to-balloon time of less than 60 or 90 minutes, even a delay of a few minutes in symptom-to-balloon time and door-to-balloon time may lead to greater infarct size and higher short- and long-term mortality.^4,5,19,20,21^ For instance, Nallamothu et al^6^ showed that every 10-minute reduction in door-to-balloon time resulted in an 8% decrease in in-hospital mortality and a 6% decrease in 6-month mortality. In our study, a mean decrease in the needle-to-balloon time of 6 minutes led to a significantly larger percentage of patients who achieved a needle-to-balloon time of 10 minutes or less (51.1% vs 19.1%; *P* < .001). Based on the aforementioned studies, it is possible that even the relatively small decrease in the needle-to-balloon time achieved in our study, which was not powered for the mortality outcome due to its small sample size, could have clinical benefit for these patients at high risk. Furthermore, while significant efforts have been made to reduce the time between the start of symptoms and arrival at the catheterization laboratory, intraprocedural techniques to shorten the needle-to-balloon time are limited.^9,10^ This study offers an additional intraprocedural technique for reducing the time to reperfusion that by itself, and to a greater extent when paired with initiatives to reduce delays before arrival at the catheterization laboratory, may improve patient outcomes.

There may be further benefits to a strategy of culprit lesion PCI before complete CAG in addition to a shorter time to reperfusion. For example, we showed that the patients who underwent CAG followed by culprit lesion PCI had a higher likelihood of crossover from a radial to a femoral approach, which, in most cases, was brought on by a spasm of the radial artery during the switch from a 5-Fr diagnostic catheter to a 6-Fr guiding catheter. However, compared with other studies of patients with STEMI that showed a crossover rate of 5.9% to 9.6%, the crossover rate in our study was higher.^22,23,24^ The need for a crossover from a radial to a femoral approach, as demonstrated by prior studies, causes an additional delay in the time to reperfusion and is associated with a higher in-hospital mortality rate.^22,23^ It is possible that the open-label design of this study has contributed to the higher crossover rate observed among the patients who underwent CAG followed by culprit lesion PCI. Larger trials are required to validate this observation and to assess the clinical effect of a crossover from a radial to a femoral approach when a culprit lesion PCI before CAG strategy is used. Also, despite no difference in the rate of vasopressors delivered, the patients who underwent culprit lesion PCI before CAG had a higher mean (SD) arterial pressure at the end of the procedure (93.6 [16.6] vs 87.6 [18.9] mm Hg; *P* = .04). This finding might be explained by a more rapid ventricular function and cardiac output recovery in this group and should be further evaluated.

Besides the advantages already mentioned, a strategy of culprit lesion PCI before CAG for patients with STEMI may have disadvantages. Some physicians can be reluctant to perform a PCI to the culprit lesion before a complete diagnostic CAG. In circumstances of inferior and/or posterior STEMI, where both the right coronary artery and left circumflex may be the culprit arteries, misidentification of the culprit lesion and a subsequent attempt to perform a PCI to a nonculprit lesion is a major drawback that could arise. In our study, the interventional cardiologist failed to correctly identify the culprit artery based on results of the electrocardiography for 9 of 90 (10.0%) patients who underwent culprit lesion PCI before CAG and for 7 of 94 patients (7.4%) who underwent CAG followed by culprit lesion PCI; moreover, for only 2 of 90 patients (2.2%) in the former group, a culprit lesion could not be identified even after complete CAG. However, a strategy of culprit lesion PCI before CAG was superior in terms of reperfusion time reduction even for patients with inferior and/or posterior STEMI, according to the subgroup analysis model used in this investigation. Moreover, attempts to perform a PCI on a nonculprit lesion were made for only 3 patients who underwent culprit lesion PCI before CAG and for 2 patients who underwent CAG followed by complete CAG. Another potential drawback of a culprit lesion PCI before CAG strategy is the possibility of performing a PCI before a full diagnostic angiography for a patient who, after a complete CAG, will be found to be a candidate for CABG. In our study, only 2 patients who underwent CAG followed by culprit lesion PCI and none of the patients who underwent culprit lesion PCI before CAG underwent urgent CABG. Moreover, based on previous studies that showed high mortality rates among patients with STEMI who were referred for urgent CABG, current society guidelines recommend that for patients with STEMI and coronary anatomy amenable for CABG (eg, patients with triple vessel disease or significant left main disease), a primary PCI to the culprit lesion should be performed, followed by an elective CABG.^25,26,27^

### Limitations

This study has some limitations. First, no clinical outcome was statistically powered in this study because of the limited sample size. To ascertain the effect of culprit lesion PCI before CAG among patients with STEMI on short-term and long-term clinical outcomes, a larger randomized clinical trial is required. Second, given the open-label design of this study, it is plausible that an interventional cardiologist’s performance bias resulted in a faster PCI and a shorter needle-to-balloon time among patients who underwent culprit lesion PCI before CAG; however, the extent of time reduction demonstrated in our study is generally comparable to that demonstrated in earlier retrospective investigations. Third, this study was a single-center investigation. The findings need to be validated by more multicenter trials.

## Conclusions

In this single-center, open-label, randomized clinical trial, culprit lesion PCI before complete CAG among patients with STEMI resulted in a shorter needle-to-balloon time and higher rates of a needle-to-balloon time of 10 minutes or less. This approach was not accompanied by higher rates of procedural or postprocedural adverse events or short-term and long-term all-cause mortality. Larger studies are needed to validate the results of this study and to assess the effect of this strategy on clinical outcomes.

## Notes

Supplement 1.
Study Protocol and Statistical Analysis Plan

Supplement 2. **eFigure.** Algorithm to Determine the Suspected Culprit Artery

Supplement 3.
Data Sharing Statement
