# Supplementary material for: Culprit Lesion Coronary Intervention Before Complete Angiography in ST-Elevation Myocardial Infarction: A Randomized Clinical Trial
Source: JAMA Netw Open. 2024 Mar 29;7(3):e243729. doi: 10.1001/jamanetworkopen.2024.3729 (PMC10980970; doi:10.1001/jamanetworkopen.2024.3729)
Supplement: Supplement 1. — Study Protocol and Statistical Analysis Plan [file jamanetwopen-e243729-s001.pdf]

Shaare Zedek Medical Center (SZMC)

Jesselson Integrated Heart Center

Study protocol:

Primary PCI to culprit artery first versus non culprit coronary artery tree demonstration following by primary PCI in Patients with ST-Elevation Myocardial Infarction

Researchers:

1. Rafael Wolff (Principal investigator)

2. Nir Levi

1. Background and Study rationale

Ischemic heart disease (IHD) is the single most common cause of death and its frequency is increasing globally.[1] It is estimated that IHD is the cause of 1.8 million deaths or 20% of all deaths in Europe.[2] Despite advancement in the fields of rapid diagnosis and in treatment strategies, the morbidity and mortality in patient with ST-segment myocardial infarction remains substantial, with an estimated mortality rate of 4-12% according to registries of the ESC countries.[3] According to the 2017 European Society of Cardiology (ESC) guidelines for the management of acute myocardial infarction in patients presenting with ST-segment elevation (STEMI), there is a level 1 recommendation with a level of evidence A for primary percutaneous coronary intervention (PPCI) strategy in patients with STEMI, and this strategy is preferred over fibrinolytic therapy.[4] In addition, the ESC guidelines recommend a first medical contact to reperfusion time within 60 minutes in STEMI patients for primary PCI-capable centers. This recommendation is supported by a recently published study that showed shortening door to balloon (D2B) time was significantly associated with survival benefit.[5] Based on the recommendation for maximal D2B time in STEMI patients to be 60 minutes, many countries and institutions worldwide have been established programs, among them the national program for quality indicators by the Israeli ministry of health, to shorten D2B times.[6] According to data published by the Israeli ministry of health regarding the year of 2018, 88% of STEMI patients had a D2B time of <90 minutes. The common practice during PPCI is to complete diagnostic angiography of the whole coronary tree before performing culprit-vessel revascularization. This practice is not evidence-based and current guidelines do not prioritize full diagnostic angiography over culprit-vessel revascularization first. As was found in previous studies, this practice might result in delaying revascularization by 4-8 minutes in D2B time.[7,8] This delay might potentially lead to worse outcome in STEMI patients, although not proven in the above cited studies.

The aim of this study is to determine the magnitude of the delay and to evaluate the clinical impact of the delay in coronary revascularization in patients with STEMI treated with PPCI.

2. Objectives

- To determine the magnitude of delay in coronary revascularization in patients with STEMI treated with PPCI after full diagnostic angiography, in comparison with patients who underwent initially coronary culprit revascularization (PPCI) following diagnostic angiography of the non-culprit coronary arteries.

- To assess the impact of diagnostic angiography of suspected culprit artery first versus full diagnostic angiography prior to coronary revascularization on D2B time.
- To assess the impact of diagnostic angiography of suspected culprit artery first versus full diagnostic angiography prior to coronary revascularization on clinical outcomes.

3. Endpoints

a. Primary endpoint:

- Reduction in Door-to-Balloon (D2B) time

  Protocol amendment:

  The investigators ask the IRB for a study protocol amendment due to reconsideration of study protocol and primary outcome:

  - **Revised primary outcome: Needle-to-balloon time within 10 minutes.**

b. Secondary endpoints:

- Need for inotropes and/or vasopressors during PCI
- Need for mechanical circulatory support (IABP, Impella or ECMO) during PCI
- Need for mechanical ventilation or non-invasive ventilation (NIV) during PCI

4. Study Cohort

- Estimated number of 300 patients (~150 each group).

a. Inclusion criteria:

- ≥18 years old
- STEMI on arrival ECG
- PPCI on arrival to SZMC

b. Exclusion criteria:

- Cardiac arrest, CPR or ECMO on arrival to catheterization laboratory
- Prior coronary artery bypass grafting (CABG) surgery

6. Methods and statistical analysis

This is a prospective, randomized, controlled, unblinded and single-center study that will include all patients with STEMI that will be admitted to SZMC. All patients will be randomized to culprit-only diagnostic angiography group (intervention arm) versus full diagnostic angiography group (control arm) prior to revascularization during PPCI on index hospitalizations. A full diagnostic angiography will be performed only after intervention to culprit artery in the intervention arm. Data will be collected from AZMA and Clicks and be held by the PI only. Baseline patients' characteristics including age, gender, cardiovascular risk factors, previous coronary interventions, current medical treatment, and comorbidities will be collected. Procedural parameters including door-to balloon time, fluoroscopy time, amount of contrast media, number of catheters and rate of conversion to femoral access will be collected and compared between the two groups. Clinical parameters including need for inotropes or vasopressors, need for mechanical circulatory support, need for mechanical ventilation or NIV, rate of ventricular tachyarrhythmias, LVEF on first in-hospital echocardiography and in-hospital mortality rate will be compared between the two groups.

Statistical analysis will be performed using SPSS Statistics for Windows (SPSS Inc., Chicago, IL). Descriptive statistics will first be used to describe the study population data. The primary and secondary endpoints will first be studied using Chi-square or Fisher's exact tests for comparisons between the outcomes for categorical variables and the t-test or Mann-Whitney U test for continuous variables. Test selection will be based on data distribution and normalcy.

Based on previously published data, the primary outcome of a needle-to-balloon time of ten minutes or less was used for sample size calculation.11–14 The calculated sample size was a minimum of 50 patients per group to detect a 50% relative increase in the proportion of patients who achieved the primary outcome (power=0.9, α=0.05, β=0.1).

7. Timetable

The study is expected to start on 1/4/2021 for a period of 12 months.

8. Ethical Issues

The researchers know of no harm or adverse effect from the change in procedure sequence between the two groups (culprit-only diagnostic angiography versus full diagnostic angiography prior to revascularization). In addition, as time is critical to the study in the unique scenario of STEMI and during primary PCI, obtaining an

informed consent might delay study intervention and treatment. Hence, a waiver of informed consent is requested from the IRB.

9. References

1. Hartley A, Marshall DC, Salciccioli JD, Sikkel MB, Maruthappu M, Shalhoub J. Trends in mortality from ischemic heart disease and cerebrovascular disease in Europe: 1980 to 2009. *Circulation*. Published online 2016. doi:10.1161/CIRCULATIONAHA.115.018931

2. Townsend N, Wilson L, Bhatnagar P, Wickramasinghe K, Rayner M, Nichols M. Cardiovascular disease in Europe: Epidemiological update 2016. *Eur Heart J*. Published online 2016. doi:10.1093/eurheartj/ehw334

3. Kristensen SD, Laut KG, Fajadet J, et al. Reperfusion therapy for ST elevation acute myocardial infarction 2010/2011: Current status in 37 ESC countries. *Eur Heart J*. Published online 2014. doi:10.1093/eurheartj/eht529

4. Ibanez B, James S, Agewall S, et al. 2017 ESC Guidelines for the management of acute myocardial infarction in patients presenting with ST-segment elevation. *Eur Heart J*. Published online 2018. doi:10.1093/eurheartj/ehx393

5. Park J, Choi KH, Lee JM, et al. Prognostic Implications of Door-to-Balloon Time and Onset-to-Door Time on Mortality in Patients With ST-Segment–Elevation Myocardial Infarction Treated With Primary Percutaneous Coronary Intervention. *J Am Heart Assoc*. Published online 2019. doi:10.1161/JAHA.119.012188

6. Nestler DM, Noheria A, Haro LH, et al. Sustaining Improvement in Door-to-Balloon Time Over 4 Years: The Mayo Clinic ST-Elevation Myocardial Infarction Protocol. *Circ Cardiovasc Qual Outcomes*. Published online 2009. doi:10.1161/CIRCOUTCOMES.108.839225

7. Plourde G, Abdelaal E, Bataille Y, et al. Effect on door-to-balloon time of immediate transradial percutaneous coronary intervention on culprit lesion in ST-elevation myocardial infarction compared to diagnostic angiography followed by primary percutaneous coronary intervention. *Am J Cardiol*. Published online 2013. doi:10.1016/j.amjcard.2012.11.059

8. Couture EL, Bérubé S, Dalery K, et al. Culprit vessel revascularization prior to diagnostic angiography as a strategy to reduce delays in primary percutaneous coronary intervention. *Circ Cardiovasc Interv*. Published

online 2016. doi:10.1161/CIRCINTERVENTIONS.115.003510

10. Appendix

A. Case report form:

Case Report Form

Primary PCI to culprit artery first versus non culprit coronary artery tree demonstration following by primary PCI in Patients with ST-Elevation Myocardial Infarction

Shaare Zedek Medical Center (SZMC)

Jesselson Integrated Heart Center

February 2021

Ver 1.4

Inclusion Criteria

- ≥18 years old

- ST-Elevation MI on arrival/MADA ECG 218
- Primary PCI on arrival to SZMC 219

Exclusion Criteria 220

- Cardiac arrest, CPR or ECMO on arrival to catheterization laboratory 221 222
- Prior CABG 223

i. Basic Information 224

1. Patient's Code _ _ _ 225
2. Patient's Group __ (1-Culprit First 2-Culprit Last) 226 227
3. ID Number _ _ _ _ _ _ _ _ _ 228
4. SZMC Accession Number _ _ _ _ _ _ _ _ _ _ _ 229
5. Date of Birth _ _/_ _/_ _ _ _ (dd/mm/yyyy) 230
6. Ethnicity __ (1-Jewish 2-Arab 3-Other) 231
7. Date of Hospital Admission _ _/_ _/_ _ _ _ 232 (dd/mm/yyyy) 233

ii. Physical Characteristics 234

1. Gender __ (1-Male 2-Female) 235
2. Weight _ _ _ (kg) 236
3. Height _ _ _ (cm) 237
4. Body Mass Index (BMI) _ _._ 238

iii. Medical History 239

1. Diabetes Mellitus __ (0-No 1-Yes) 240
2. Dyslipidemia __ (0-No 1-Yes) 241
3. Hypertension __ (0-No 1-Yes) 242
4. Congestive Heart Failure (CHF) __ (0-No 1-Yes) 243
5. Active Smoker __ (0-No 1-Yes) 244
6. Past Smoker __ (0-No 1-Yes) 245
7. Renal Failure __ (0-No 1-Yes) 246
8. Peripheral Vascular Disease __ (0-No 1-Yes) 247
9. Family History of CAD __ (0-No 1-Yes) 248
10. Atrial Fibrillation __ (0-No 1-Yes) 249
11. Prior Stroke __ (0-No 1-Yes) 250
12. Prior CAD __ (0-No 1-Yes) 251
13. Prior PCI __ (0-No 1-Yes) 252
14. Prior CABG __ (0-No 1-Yes) 253
15. Prior Valve Surgery __ (0-No 1-Yes) 254
16. Prior TAVR __ (0-No 1-Yes) 255

iv. Baseline Medical Treatment 256

1. Antiplatelet Therapy __ (0-No 1-Aspirin 2-Clopidogrel 3-Ticagrelor 4-Prasugrel 5-DAPT 99-Unknown) 257 258 259
2. Anticoagulation Therapy __ (0-No 1-Apixaban 2-Rivaroxaban 3-Dabigatran 4-VKA 5-LMWH 6-Heparin 99-Unknown) 260 261 262
3. Beta-Blocker __ (0-No 1-Yes) 263
4. ACE Inhibitor/ARB __ (0-No 1-ACEI 2-ARB) 264
5. Statin Therapy __ (0-No 1-Yes) 265
6. PCSK9 __ (0-No 1-Yes) 266
7. Calcium-Channel Blocker __ (0-No 1-Yes) 267
8. Loop Diuretics __ (0-No 1-Yes) 268
9. Thiazide __ (0-No 1-Yes) 269
10. K-Sparing Diuretic __ (0-No 1-Yes) 270

11. Anti-Diabetic Treatment __ (0-No 1-Yes) 271
12. Anti-Arrhythmic Therapy __ (0-No 1-Yes) 272

v. Laboratory Tests (First, Last, Max and Min Values) 273

1. White Blood Count (WBC) _ _._ (10^3/µL) 274
2. Platelets (PLT) _ _ _ (10^3/µL) 275
3. Hemoglobin (Hb) _ _._ (g/dL) 276
4. Potassium _._ (mEq/L) 277
5. Blood Urea Nitrogen (BUN) _ _ _ (mg/dL) 278
6. Creatinine _ _._ _ (mg/dL) 279
7. Lactic Acid Dehydrogenase (LDH) _ _ _ (IU/L) 280
8. Lactate _ _._ (mmol/L) 281
9. High-Sensitive cTnI _ _ _ _ _ _ (ng/L) 282
10. Creatine Phosphokinase (CPK) _ _ _ _ _ (IU/L) 283
11. Prothrombin time (PT) _ _ ._ (sec) 284
12. Partial Thromboplastin Time (PTT) _ _._ (sec) 285
13. International Normalized Ratio (INR) _ _._ 286
14. Fibrinogen _ _ _ _ (mg/dL) 287

vi. Procedural Data 288

1. MI Type __ (1-Anterior 2-Anterior-Lateral 3-Lateral 4-Inferior 5-Inferior-Posterior 6-Posterior 7-Diffused ST-Depression 88-Undetermined) 289 290 291 292
2. Day of the Week __ (1-7) 293
3. Arrival to Cath Lab __ (1-Directly by MADA 2-From ER 3-Other) 294 295
4. Hour at Arrival to Cath Lab _ _:_ _ (hh:mm) 296
5. First Medical Contact Time (FMCT) __ (hh:mm) 297 298
6. Initial Heart Rate _ _ _ (bpm) 299
7. Initial Systolic Blood Pressure _ _ _ (mmHg) 300
8. Initial Diastolic Blood Pressure _ _ _ (mmHg) 301
9. Initial Saturation _ _ (%) 302
10. Assumed Side of Culprit Artery __ (1-Right System 2-Left System 3-Undetermined) 303 304
11. Time of cath initiation _ _:_ _ (hh:mm) 305
12. Needle Time _ _:_ _ (hh:mm) 306
13. Reperfusion Time _ _:_ _ (hh:mm) 307
14. End-Case Time _ _:_ _ (hh:mm) 308
15. Access Site __ (1-Rt Radial 2-Lt Radial 3-Rt Femoral 4-Lt Femoral 88-Other) 309 310
16. Access Site Conversion __ (0-No 1-Yes) 311
17. Culprit Artery __ (1-LM 2-LAD 3-Ramus 4-LCx 5-RCA 88-Undetermined) 312 313
18. Significant LM Disease __ (0-No 1-Yes) 314
19. Significant LAD Disease __ (0-No 1-Yes) 315
20. Significant Ramus Disease __ (0-No 1-Yes) 316
21. Significant LCx Disease __ (0-No 1-Yes) 317
22. Significant RCA Disease __ (0-No 1-Yes) 318
23. PCI to LM __ (0-No 1-Yes) 319
24. PCI to LAD __ (0-No 1-Yes) 320
25. PCI to Ramus __ (0-No 1-Yes) 321
26. PCI to LCx __ (0-No 1-Yes) 322
27. PCI to RCA __ (0-No 1-Yes) 323
28. Total Number of Stents Deployed __ (0-9) 324
29. Total Number of Wires Used __ (0-9) 325

30. Total Number of Diagnostic/Guiding Catheters Used __ (0-9) 326 327
31. IC Imaging __ (0-No 1-IVUS 2-OCT) 328
32. FFR/IFR Use __ (0-No 1-Yes) 329
33. IIB/IIIA inhibitors administration __ (0-No 1-Yes) 330 331
34. Total Fluoroscopic Time _ _ _ (Min) 332
35. Total Contrast Delivered _ _ _ (mL) 333
36. End-Procedural Heart Rate _ _ _ (bpm) 334
37. End-Procedural Systolic Blood Pressure _ _ _ (mmHg) 335 336
38. End-Procedural Diastolic Blood Pressure _ _ _ (mmHg) 337 338
39. End-procedural Saturation _ _ (%) 339
40. Door-to-Needle Time __ (Min) 340
41. Needle-to-Balloon __ (Min) 341
42. Door-to-Balloon __ (Min) 342
43. FMCT-to-Balloon Time __ (Min) 343

vii. Intra-Procedural Complications 344

1. Coronary Dissection __ (0-No 1-Yes) 345
2. Coronary Perforation __ (0-No 1-Yes) 346
3. No-Reflow __ (0-No 1-Yes) 347
4. Failed PCI __ (0-No 1-Yes) 348
5. Urgent CABG __ (0-No 1-Yes) 349
6. Ischemic Stroke __ (0-No 1-Yes) 350
7. Blood Products Administration __ (0-No 1-Yes) 351 352
8. Inotropes/Vasopressors Administration __ (0-No 1-Yes) 353 354
9. Ventricular Arrhythmia __ (0-No 1-Yes) 355
10. DC Cardioversion __ (0-No 1-Yes) 356
11. Temporary Pacing __ (0-No 1-Yes) 357
12. Permanent Pacing __ (0-No 1-Yes) 358
13. PEA/Asystole __ (0-No 1-Yes) 359
14. Non-Invasive Ventilation __ (0-No 1-Yes) 360
15. Mechanical Ventilation __ (0-No 1-Yes) 361
16. Mechanical Complication __ (0-No 1-Acute MR 2-Ventricular Septal Rupture 3-LV Free Wall Rupture) 362 363 364
17. Mechanical Circulatory Support __ (0-No 1-IABP 2-Impella 3-ECMO) 365 366
18. Death __ (0-No 1-Yes) 367

viii. Post-Procedural Complications 368

1. Access Site Bleeding __ (0-No 1-Yes) 369
2. Retroperitoneal Hemorrhage __ (0-No 1-Yes) 370
3. Ischemic Stroke __ (0-No 1-Yes) 371
4. Blood Products Administration __ (0-No 1-Yes) 372 373
5. Initiation of Hemodialysis __ (0-No 1-Yes) 374
6. Inotropes/Vasopressors Administration __ (0-No 1-Yes) 375 376
7. Ventricular Arrhythmia __ (0-No 1-Yes) 377
8. DC Cardioversion __ (0-No 1-Yes) 378
9. Temporary Pacemaker Insertion __ (0-No 1-Yes) 379 380

10. PEA/Asystole __ (0-No 1-Yes) 381
11. Non-Invasive Ventilation __ (0-No 1-Yes) 382
12. Mechanical Ventilation __ (0-No 1-Yes) 383
13. Mechanical Complication __ (0-No 1-Acute 384
MR 2-Ventricular Septal Rupture 3-LV Free 385
Wall Rupture) 386
14. Mechanical Circulatory Support __ (0-No 1- 387
IABP 2-Impella 3-ECMO) 388
15. In-Hospital Death __ (0-No 1-Yes) 389
16. 30-Day All-Cause Death __ (0-No 1-Yes) 390

ix. Pre-Discharge Echocardiography 391

1. Left-Ventricular Ejection Fraction _ _ (%) 392
2. Grade of Mitral Regurgitation __ (0-No MR 1- 393
Mild MR 2-Mild-Moderate MR 3-Moderate 394
MR 4-Moderate-Severe MR 5-Severe MR) 395
3. Apical Thrombus __ (0-No 1-Yes) 396

x. Hospitalization Information 397

1. Monitoring Level at Admission to ICCU __ (1- 398
Level I 2-Level IIa 3-Level IIb 4-Level III) 399
2. Length of Hospitalization in ICCU __ (days) 400
3. Length of Hospitalization in Cardiology Dept. 401
__ (days) 402
4. Total Length of Hospitalization __ (days) 403
5. In-Hospital CABG __ (0-No 1-Yes) 404
6. Redo Cardiac Cath __ (0-No 1-Yes) 405

406

407

408

409

410

411

412

413

414

415

416
