# Supplementary material for: Culprit Lesion Coronary Intervention Before Complete Angiography in ST-Elevation Myocardial Infarction: A Randomized Clinical Trial
Source: JAMA Netw Open. 2024 Mar 29;7(3):e243729. doi: 10.1001/jamanetworkopen.2024.3729 (PMC10980970; doi:10.1001/jamanetworkopen.2024.3729)
Supplement: Supplement 2. — eFigure. Algorithm to Determine the Suspected Culprit Artery [file jamanetwopen-e243729-s002.pdf]

Supplementary Online Content

Levi N, Wolff R, Jubeh R, et al. Culprit lesion coronary intervention before complete angiography in ST-elevation myocardial infarction: a randomized clinical trial. *JAMA Netw Open.* 2024;7(3):e243729. doi:10.1001/jamanetworkopen.2024.3729

eFigure. Algorithm to Determine the Suspected Culprit Artery

This supplementary material has been provided by the authors to give readers additional information about their work.

© 2024 Levi N et al. *JAMA Network Open.*

eFigure. Algorithm to Determine the Suspected Culprit Artery

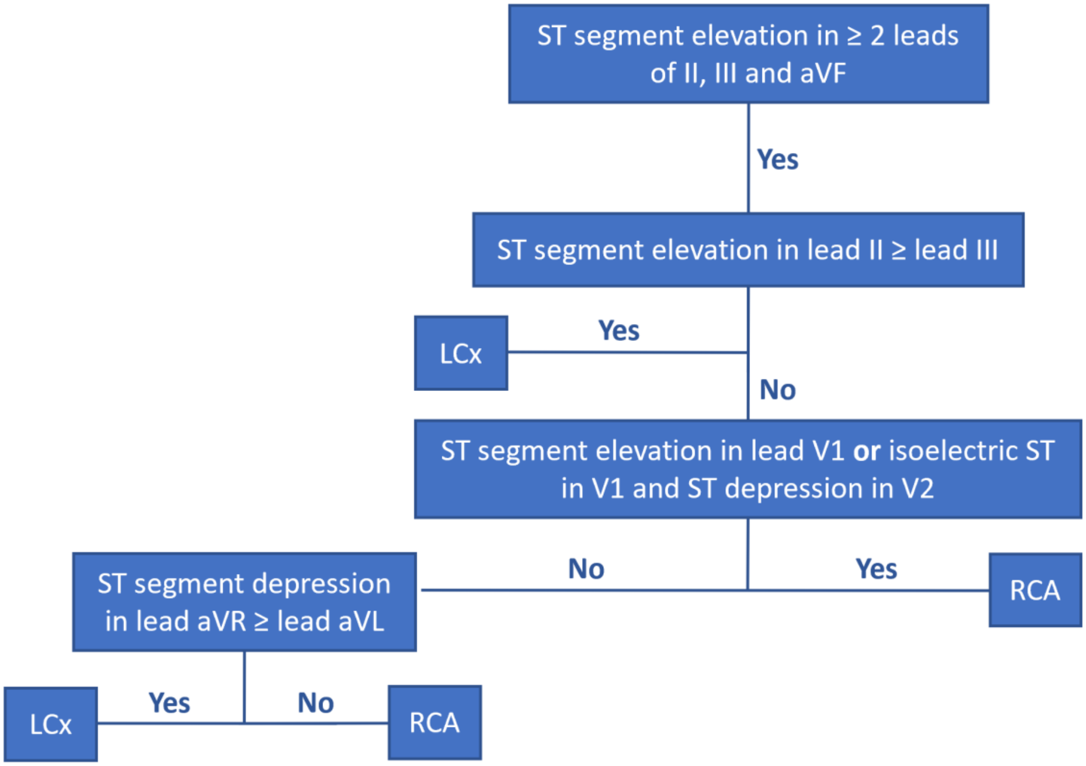

© 2024 Levi N et al. *JAMA Network Open.*
