# Supplementary material for: Culprit Lesion Coronary Intervention Before Complete Angiography in ST-Elevation Myocardial Infarction: A Randomized Clinical Trial
Source: JAMA Netw Open. 2024 Mar 29;7(3):e243729. doi: 10.1001/jamanetworkopen.2024.3729 (PMC10980970; doi:10.1001/jamanetworkopen.2024.3729)
Supplement: Supplement 3. — Data Sharing Statement [file jamanetwopen-e243729-s003.pdf]

Data Sharing Statement

Levi. Culprit Lesion Coronary Intervention Before Complete Angiography in ST-Elevation Myocardial Infarction. *JAMA Netw Open*. Published March 29, 2024.
doi:10.1001/jamanetworkopen.2024.3729

Data
Data available: No

Additional Information
Explanation for why data not available: Data will be published in later stages.
